# Chord Mu (µ) and Chord Alpha (α) Length Changes in Fuchs Endothelial Corneal Dystrophy before and after Descemet Membrane Endothelial Keratoplasty (DMEK) Surgery

**DOI:** 10.3390/jcm10214844

**Published:** 2021-10-21

**Authors:** Carlos Rocha-de-Lossada, José-María Sánchez-González, Davide Borroni, Víctor Llorens-Bellés, Rahul Rachwani-Anil, Josep Torras-Sanvicens, Vito Romano, Jorge Peraza-Nieves

**Affiliations:** 1 Department of Ophthalmology, Hospital Clinic de Barcelona Institut Clinic d’Oftalmologia, 08036 Barcelona, Spain; carlosrochadelossada5@gmail.com (C.R.-d.-L.); llorens.victor@gmail.com (V.L.-B.); jts29206@gmail.com (J.T.-S.); jorge.peraza.nieves@gmail.com (J.P.-N.); 2 Department of Ophthalmology (Qvision), VITHAS Hospital, 04120 Almería, Spain; 3 Department of Ophthalmology, Hospital Virgen de las Nieves, 18014 Granada, Spain; 4 Department of Physics of Condensed, Optics Area, University of Seville, 41012 Seville, Spain; 5 Department of Ophthalmology (Tecnolaser Clinic Vision®), Refractive Surgery Centre, 41018 Seville, Spain; 6 Department of Doctoral Studies, Riga Stradins University, LV-1007 Riga, Latvia; info.borroni@gmail.com (D.B.); vito.romano@gmail.com (V.R.); 7 Department of Ophthalmology, Royal Liverpool University Hospital, Liverpool L7 8XP, UK; 8 Department of Ophthalmology, Antequera Hospital, 29200 Malaga, Spain; rahul.medum@gmail.com

**Keywords:** chord mu (µ), chord alpha (α), kappa angle, alpha angle, Descemet membrane endothelial keratoplasty, DMEK, multifocal intraocular lens

## Abstract

This paper will evaluate chord mu and alpha length in patients with Fuchs endothelial corneal dystrophy (FECD) and its changes following Descemet membrane endothelial keratoplasty (DMEK). Patients with FECD that underwent DMEK surgery were included in this retrospective study. Scheimpflug Tomography was carried out in order to calculate chord mu and chord alpha lengths prior to surgery and at 3 and 12 months postoperative. This study included 27 eyes from 27 patients. Significant changes in chord mu were observed within the first three months (from 0.47 ± 0.32 to 0.29 ± 0.21 mm, *p* < 0.01) and remained stable 12 months postoperative (0.30 ± 0.21 mm, *p* > 0.05). However, chord alpha remained stable throughout the 12 months post surgery (from 0.53 ± 0.19 to 0.49 ± 0.14 mm, *p* > 0.05). In addition to the pupillary center distance from the corneal center (from 0.35 ± 0.25 to 0.34 ± 0.20 mm, *p* > 0.05) also remain stable. In FECD patients undergoing DMEK surgery, chord mu length decreased, and chord alpha length remained stable after 12 months of follow-up.

## 1. Introduction

Descemet membrane endothelial keratoplasty (DMEK) is the main endothelial transplantation technique [1,2,3]. DMEK has proven to attain better results in visual acuity and a faster recovery compared to other corneal transplant techniques [4,5,6]. Recently, Birbal et al. [7] reported that after five years of follow-up after DMEK surgery, graft survival is high, visual acuity outcomes are excellent, and there is a low long-term complication rate. Nanavaty et al. [8] reported a patient with Fuchs endothelial corneal dystrophy (FECD) who provided successful results after a combined refractive lens exchange with a multifocal intraocular lens (MIOL) in addition to DMEK. Moreover, other cases of DMEK after corneal decompensation a few years after MIOL implantation in eyes with FECD have been described [9].

Currently, the number of people undergoing MIOL implantation has progressively increased [10]. A large deviation between the visual axis and the pupillary axis of the MIOL can lead to higher-order aberrations postoperatively, resulting in decreased visual quality [10]. Therefore, some authors propose including the measurement of chord mu and chord alpha in preoperative examinations in patients scheduled for MIOL implantation [10,11,12]. Chord mu is defined as the distance from the pupil center (line of sight) to the light reflex (topographer axis) [13], and it has been described that it is possible to measure it accurately with a Pentacam Comprehensive Eye Scanner (Oculus Optikgeraete GmbH; Wetzlar, Germany) [13]. Values above 0.4–0.6 mm have been associated with the presence of halos or glare with diffractive MIOLs [14,15]. Chord alpha was defined as the distance between the corneal center and the corneal vertex. Fu et al. [10] and Piracha [11] found that if the distance of the chord alpha is larger than 0.5 mm, the eye will not be suitable for MIOL implantation. Recently, Fernández et al. [16] have studied how biometric factors could be linked to visual performance in high addition MIOL and found that chord mu was significantly reduced after cataract surgery. Similar results were reported by Wang et al. [17].

The aim of this study was to describe chord mu and chord alpha in patients affected by FECD and, moreover, describe longitudinal changes in these parameters during the first year after DMEK surgery.

## 2. Materials and Methods

In this retrospective study, all DMEK surgeries performed at the Hospital Clinic of Barcelona in patients suffering from FECD between March 2017 and March 2019 by the same surgeon with at least 12 months of follow-up were included. All patients were pseudophakic. Data prior to surgery, and at 1, 3, and 12 months postoperative were collected. Patients with previous corneal surgery, iris defects different from YAG-iridotomy, and intra/postoperative complications were excluded. The retrospective data collection followed the tenets of the Declaration of Helsinki and was approved by the local Institutional Review Board.

**Figure 1:**
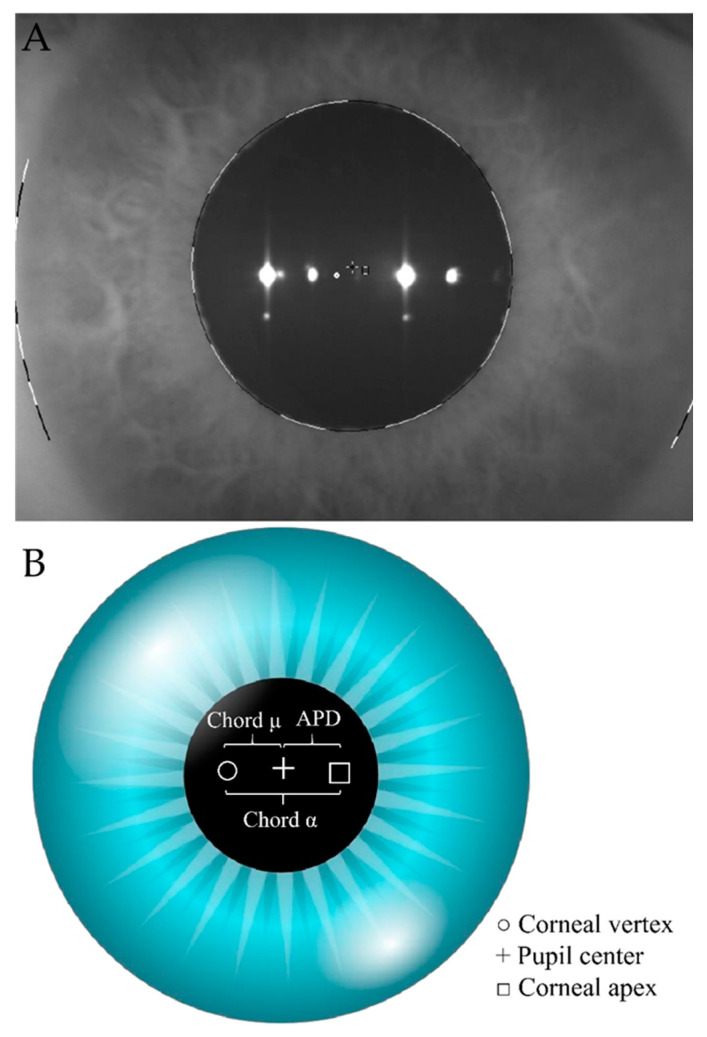
(**A**)—real left eye iris map from Scheimpflug Tomography (Pentacam HR; Oculus, Wetzlar, Germany). To measure chord alpha (α) length, we pointed to the center of the square (□) which corresponded to the center of the cornea, and we joined it to the center of the white circle (○) which corresponded to the corneal vertex. The distance in millimeters (mm.) between both points was defined as chord alpha (α) length. To measure APD, we joined the corneal center (□) to the center of the black cross (+) which corresponded to the center of the pupil. The distance in millimeters (mm.) between both points was defined as APD. (**B**)—simulated iris map from Scheimpflug Tomography (Pentacam HR; Oculus, Wetzlar, Germany). The white circle (○) corresponds to the corneal vertex. The black cross (+) corresponds to the center of the pupil and the square (□) corresponds to the center of the cornea. APD: Corneal center to pupil center.

DMEK graft was prestripped at the Barcelona Eye Bank and sent to our hospital in organic culture medium (CorneaMax; Eurobio, Les Ulis, France). All DMEK procedures were performed by the same surgeon (JPN) under local anesthetic following the Melles technique [18]. Chord mu was defined as the distance in millimeters (mm) from the pupil center (line of sight) to the light reflex (topographer axis). Chord alpha was defined as the distance in mm between corneal center and corneal vertex. The distance in mm between corneal center and pupil center distance (APD) was also measured to better assess pupil center stability (Figure 1B). Both measurements were extracted from the iris map of the Scheimpflug camera (Pentacam HR; Oculus, Wetzlar, Germany) (Figure 1A). Moreover, central corneal thickness (CCT), corneal center thickness (CAT), and thinnest point (THP) were measured with a Scheimpflug camera (Pentacam HR; Oculus, Wetzlar, Germany). All the examinations were performed under scotopic light conditions by the same operator (CRL). All data were accepted as high quality by the Pentacam quality control system. Corrected distance visual acuity (CDVA) was measured with a 20 feet Snellen chart in photopic conditions in each revision. Chord mu (including X and Y coordinates and their meridian in degrees) were extracted from topometric/keratoconus staging Pentacam map. Coordinate origin (0,0) measurements were black cross center (+).

APD and chord alpha measurements were extracted from the iris map of the Pentacam (Figure 1). To measure chord alpha, we pointed in the center of the square (□) which corresponded to the center of the cornea, and we joined it to the center of the white circle (○) which corresponded to the corneal vertex. The distance in mm between both points was defined as the length of chord alpha. To measure APD, we joined the corneal center (□) to the center of the black cross (+) which corresponded to the center of the pupil. The distance in mm between both points was defined as APD. All measurements were performed under the same scotopic room illumination.

### Statistical Analysis

Data were analyzed with SPSS statistics software (version 26.0 for Windows; SPSS Inc., Chicago, IL, USA). Descriptive analysis was carried out with values expressed as mean ± standard deviation and range. Normality distribution was assessed with Shapiro–Wilk test. Longitudinal differences in mean values were assessed with Student’s *t*-test or Wilcoxon test. False Discovery Rate (FDR) was analyzed with the Benjamini–Hochberg test. For all tests, the level of significance was established at 95% (*p* < 0.05). Accepting an alpha risk of 0.05 and beta risk of 0.2 in a two-sided test, 25 subjects were necessary to recognize a difference greater than or equal to 0.05 as statistically significant. The standard deviation was assumed to be 0.15. A drop-out rate of 10% was anticipated.

## 3. Results

Twenty-seven eyes from twenty-seven Caucasian patients were included in this study. The mean age was 75.4 ± 12.1 years old (from 52 to 91 years). Eighteen females and nine males were included. Nineteen were right eyes and eight were left eyes. The button size was 8.29 ± 0.25 (8.00 to 8.50). Table 1 summarizes all the study outcomes at baseline and at 12 months of follow-up. No significant differences were observed in chord mu length in 12 months of follow-up, that varied from 0.47 ± 0.32 mm (X: −0.14 ± 0.37 and Y: −0.16 ± 0.40 in 198.24 ± 93.98 meridian degrees) to 0.30 ± 0.21 mm (X: −0.03 ± 0.24 (SD) and Y: −0.05 ± 0.33 (SD) in 200.11 ± 96.96 meridian degrees, *p* = 0.75). 

**Figure 2:**
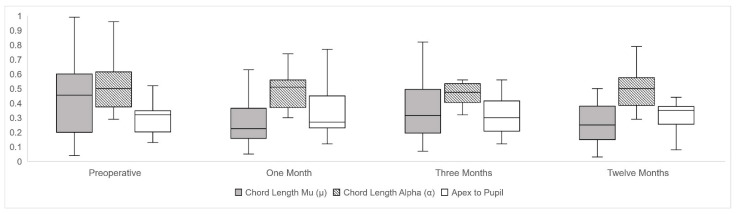
Longitudinal changes of chord length mu (µ), chord length alpha (α) and center to pupil distance (APD) along preoperative and postoperative follow-up represented by box and plot graphs, expressed in millimeters (mm).

A second analysis was carried out, separating the right and left eyes [19], and similarly, no statistically significant differences were obtained in the X and Y coordinates and in the meridian degrees in the chord mu length. Significant changes were found in chord mu prior to surgery compared to one month postoperative (*p* < 0.01), and between three months and one year (*p* < 0.05) of follow-up. There were no significant differences between preoperative and twelve months postoperative in chord mu meridian orientation. Chord alpha did not vary during the follow-up. APD did not suffer any changes from 0.35 ± 0.24 mm to 0.34 ± 0.20 mm (*p* = 0.52) between preoperative and twelve months postoperative after DMEK. Longitudinal evolution changes of chord mu, chord alpha, and APD were reported using box and plot graphs in Figure 2.

## 4. Discussion

The study of chord mu and chord alpha has proven to be important in surgeries undergoing MIOL implantation [20,21], as it could predict postoperative complications such as halos, glare, or dysphotopsias [10]. Currently, DMEK has proved to attain excellent results regarding visual acuity and refractive state [22,23]. Moreover, Chaurasia et al. [24] reported visual outcomes and surgical complications of DMEK versus simultaneous IOL implantation and DMEK combined in 429 patients with FECD. They concluded that a simultaneous combined procedure was not linked with any high-risk complication versus DMEK alone. In addition, they suggested that combined surgery is an effective strategy in rapid visual rehabilitation and may offer the advantage of a one-stage procedure, as well as a reduced cost. Patients undergoing DMEK may be potential candidates for MIOLs favored by the speed of recovery and postoperative stability.

Pereira et al. [9] reported a case series of DMEK as a secondary procedure after corneal decompensation a few years after MIOL implantation. This group described good outcomes after DMEK surgery in all patients. Similarly, Price et al. [25] recently described the outcomes of 14 cases with extended depth of focus IOLs and two bifocal IOLs implantation in 16 eyes of eight FECD patients who reached satisfactory refractive and visual outcomes after cataract surgeries previously operated by DMEK surgery.

Therefore, a study of chord mu and chord alpha in the postoperative period of DMEK surgery seems to be necessary to assess the potential implantation of MIOLs in these patients. Moreover, little is known regarding chord mu and chord alpha in FECD. The data presented in this current study indicated that chord mu reduced significantly during the first month after surgery and remained stable after 12 months of follow-up. However, it did not attain statistically significant differences at three and twelve months of follow-up versus preoperative values. This fact may be due to the relatively small sample size. Conversely, chord alpha and APD did not achieve significant differences in the postoperative follow-up. As expected, corneal thickness parameters (CCT, CAT, and THP) were significantly reduced from preoperative values compared to all postoperative appointments. According to our outcomes, previous corneal edema could suggest a visual axes misalignment. Therefore, after corneal clearance, the visual axis may be realigned. Consequently, chord mu significantly reduced after the first month and remained stable for at least 12 months of follow-up. Hashemi et al. [26] using the Orbscan II device in a population study of 800 eyes with a large age distribution (40.6 ± 16.8, range 14–81 years), found a mean value of angle kappa of 5.13 ± 1.50° (myopes), 5.72 ± 1.10° (emmetropes), and 5.52 ± 1.19° (hyperopes). However, according to Holladay [13] the mean chord mu measurements on the Scheimpflug device are 0.20 ± 0.11 mm, hence the upper limit of the normal range at a 95% CI for actual chord mu would be 0.42 mm. Here, we found a preoperative mean chord mu of 0.47 ± 0.32, which is larger than that reported by Holladay [13]. We hypothesize that, due to the preoperative corneal edema that exists in FECD patients, this chord mu could be higher than the normal population due to a possible misalignment of the visual axis. However, in the postoperative period, when the corneal thickness is decreased and the corneal clearance is achieved, we observed how chord mu decreased and reached values quite similar to those reported in the normal population.

Mahr et al. [27] established alpha angle normative values in 3382 eyes. They concluded that alpha angle magnitude was within 0.44 ± 0.15 mm. We found a mean preoperative chord alpha value of 0.53 mm in our FECD patients, which is quite similar to those reported by Mahr [27]. However, and unlike them, we used the chord alpha instead of the alpha angle. Likewise, recently Wang et al. [17] demonstrated that there were no significant changes between preoperative and postoperative angle alpha after cataract surgery, unlike in the kappa angle which significantly decreased. This is similar to our results where we found that chord alpha remained unchanged and chord mu decreased after DMEK. Again, as we used Pentacam^®^ as a topograph and we used the term chord mu. Regarding APD, our findings showed no significant differences in APD between previous and postoperative follow-up. This could be explained as APD measures the corneal structural distance between center and pupil.

In order to avoid any pupillary changes, all DMEK had preoperative iridotomy prior to basal measurements and we used air tamponade instead of gas in order to avoid increased IOP damaging the pupillary sphincter, assuring no morphological changes over the pupil [28,29]. Here, we found no statistically significant differences in the X and Y coordinates and in the meridian degrees in chord mu length, suggesting that the pupil remained in the same place, at least in our sample and with our surgical technique.

Longitudinal changes in chord mu, chord alpha, or APD could be future lines of research after other surgeries. Descemet stripping-automated endothelial keratoplasty (DSAEK), intracorneal ring segments (ICRS) or laser assisted in situ keratomileusis (LASIK) [30] could be possible candidates for MIOLs. Alpha and kappa angles or chords are of importance, besides in the preoperative study of the MIOL [10,31]. Traditionally, it has been emphasized that a larger kappa angle could negatively affect the subsequent satisfaction of patients with MIOL or refractive surgery [12,20,21,32], although this fact is currently under debate [33]. Actually, the kappa angle or chord mu could change after surgery [16,17,34]. Recently, the role of the alpha angle or chord alpha seems to take more importance. Unlike, chord mu, chord alpha could stay relatively stable after the procedure [17,35,36]. Therefore, according to us, more evidence is still necessary. Since there are already studies which report the use of MIOLs [9,25] in DMEK, we consider that gaining more knowledge regarding the behavior of both chords, mu, and chord alpha after corneal endothelial transplantation could be useful for the scientific community. However, the role of both in the possible influence in a combined surgery of DMEK and MIOL or refractive surgery must be studied in the future, since our purpose here was only to report how both could vary after DMEK. Likewise, factors such as previous higher-order aberrations after endothelial keratoplasty may be important for successful outcomes following MIOL add-on implantation. Similarly, studying longitudinal morphological changes in chord mu and chord alpha using different tamponades besides air, such as sulfur hexafluoride or perfluropropane, could be interesting.

The retrospective design and a small sample size are the main limitations of our study. Another limitation was the lack of data regarding refractive patients’ status of some patients. This fact may influence in the amount of both chords. However, although the largest pupil offset could be found in the hyperopic normal patients, recently it was shown that there was also a wide range of pupil offset in myopic and emmetropic eyes [16]. Nevertheless, on the one hand, due to lack of data of some of them—since they were derived from other centers—and in the other hand, because refraction may not be reliable due to preoperative corneal edema status in some of them, we decided not to include the refraction status here. Furthermore, our main objective was to analyze how both cords could vary between preoperative and postoperative after DMEK surgery, and not how they correlated with the previous refractive error. This could be an object of study for future work. Regarding our strengths, all surgeries were performed by one senior surgeon using the same tamponade in all surgeries, and all the measurements were carried out by one single operator with the same conditions. To the best of our knowledge, this is the first report that describes chord mu and chord alpha in FECD patients and describes their changes throughout the first year following DMEK surgery.

## 5. Conclusions

In conclusion, chord mu decreased in the first month after DMEK and remained stable with minimal variation, especially between the third and twelfth month. Furthermore, chord alpha remained unchanged after surgery and during the entire follow-up. Here, we describe the behavior of these variables after DMEK surgery. These findings may be necessary to consider in patients planning for MIOL implantation after combination with DMEK surgery.

## Figures and Tables

**Table 1:** Descriptive analysis represented by mean ± standard deviation (range).

Variable	Preoperative and *p* Value	One Month	Three Months	Twelve Months
Chord length mu Coordinate (X) *	−0.14 ± 0.37 (−0.98 to +0.44)	−0.09 ± 0.25 (−0.79 to +0.39)	−0.11 ± 0.25 (−0.65 to +0.37)	−0.03 ± 0.24 (−0.48 to +0.50)
Chord length mu Coordinate (Y) *	−0.16 ± 0.40 (−1.36 to +0.41)	−0.04 ± 0.24 (−0.53 to +0.59)	0.01 ± 0.36 (−0.89 to +0.72)	−0.05 ± 0.33 (−0.87 to +0.73)
Chord length mu Meridian (°) *	198.24 ± 93.82 (8.50 to 356.00)	174.29 ± 88.62 (1.90 to 295.10)	175.86 ± 81.39 (35.40 to 313.80)	200.11 ± 96.96 (36.90 to 326.00)
Chord length mu Distance (mm)	0.47 ± 0.32 (0.04 to 1.36)	0.29 ± 0.21 (0.05 to 0.95)	0.37 ± 0.25 (0.07 to 1.10)	0.30 ± 0.21 (0.03 to 0.99)
	*p* (Pre vs.)	<0.01 *	0.10	0.06
	*p* (1 month vs.)		0.09	0.17
	*p* (3 months vs.)			<0.05 *
Chord length alpha Distance (mm)	0.53 ± 0.19 (0.29 to 0.96)	0.51 ± 0.17 (0.30 to 0.96)	0.50 ± 0.19 (0.21 to 1.05)	0.49 ± 0.14 (0.29 to 0.79)
	*p* (Pre vs.)	0.60	0.71	0.05
	*p* (1 month vs.)		0.69	0.55
	*p* (3 months vs.)			0.94
APD Distance (mm)	0.35 ± 0.24 (0.13 to 1.07)	0.33 ± 0.16 (0.12 to 0.77)	0.31 ± 0.14 (0.12 to 0.56)	0.34 ± 0.20 (0.08 to 0.89)
	*p* (Pre vs.)	0.67	0.29	0.52
	*p* (1 month vs.)		0.35	0.57
	*p* (3 months vs.)			0–66
CCT (µm)	705.19 ± 92.73 (505.00 to 875.00)	547.67 ± 58.82 (439.00 to 666.00)	544.52 ± 79.95 (430.00 to 766.00)	555.95 ± 66.95 (462.00 to 786.00)
	*p* (Pre vs.)	<0.01 *	<0.01 *	<0.01 *
	*p* (1 month vs.)		0.54	0.55
	*p* (3 months vs.)			0.38
CAT (µm)	697.48 ± 93.11 (506.00 to 893.00)	555.80 ± 66.06 (437.00 to 708.00)	548.65 ± 88.66 (428.00 to 833.00)	560.40 ± 70.65 (463.00 to 808.00)
	*p* (Pre vs.)	<0.01 *	<0.01 *	<0.01 *
	*p* (1 month vs.)		0.82	0.42
	*p* (3 months vs.)			0.50
THP (µm)	655.11 ± 78.81 (503.00 to 826.00)	532.12 ± 53.30 (430.00 to 639.00)	529.26 ± 65.92 (418.00 to 669.00)	540.35 ± 46.10 (455.00 to 636.00)
	*p* (Pre vs.)	<0.01 *	<0.01 *	<0.01 *
	*p* (1 month vs.)		0.93	<0.05 *
	*p* (3 months vs.)			0.15

APD: Corneal center to pupil center distance; CCT: Corneal central thickness; CAT: Corneal center thickness; THP: Corneal thickness thinnest point. * All *p* values were >0.05.

## Data Availability

Data available on request due to restrictions.

## References

[1] Flockerzi E., Maier P., Böhringer D., Reinshagen H., Kruse F., Cursiefen C., Reinhard T., Geerling G., Torun N., Seitz B. (2018). Trends in Corneal Transplantation from 2001 to 2016 in Germany: A Report of the DOG–Section Cornea and its Keratoplasty Registry. Am. J. Ophthalmol..

[2] Borroni D., Gadhvi K., Wojcik G., Pennisi F., Vallabh N.A., Galeone A., Ruzza A., Arbabi E., Menassa N., Kaye S. (2020). The Influence of Speed during Stripping in Descemet Membrane Endothelial Keratoplasty Tissue Preparation. Cornea.

[3] Parekh M., Borroni D., Ruzza A., Levis H.J., Ferrari S., Ponzin D., Romano V. (2018). A comparative study on different Descemet membrane endothelial keratoplasty graft preparation techniques. Acta Ophthalmol..

[4] Singh A., Zarei-Ghanavati M., Avadhanam V., Liu C. (2017). Systematic Review and Meta-Analysis of Clinical Outcomes of Descemet Membrane Endothelial Keratoplasty Versus Descemet Stripping Endothelial Keratoplasty/Descemet Stripping Automated Endothelial Keratoplasty. Cornea.

[5] Parekh M., Leon P., Ruzza A., Borroni D., Ferrari S., Ponzin D., Romano V. (2018). Graft detachment and rebubbling rate in Descemet membrane endothelial keratoplasty. Surv. Ophthalmol..

[6] Stuart A.J., Romano V., Virgili G., Shortt A.J. (2018). Descemet’s membrane endothelial keratoplasty (DMEK) versus Descemet’s stripping automated endothelial keratoplasty (DSAEK) for corneal endothelial failure. Cochrane Database Syst. Rev..

[7] Birbal R.S., Ni Dhubhghaill S., Bourgonje V.J.A., Hanko J., Ham L., Jager M.J., Böhringer S., Oellerich S., Melles G.R.J. (2020). Five-Year Graft Survival and Clinical Outcomes of 500 Consecutive Cases After Descemet Membrane Endothelial Keratoplasty. Cornea.

[8] Nanavaty M.A., Ashena Z. (2020). Refractive lens exchange with a trifocal intraocular lens in Fuchs endothelial dystrophy. J. Cataract Refract. Surg..

[9] Pereira N.C., Diniz E.R., Ghanem R.C., Filho R.C., Prazeres T.M., Nose W., Forseto A. (2018). dos S. Descemet membrane endothelial keratoplasty in multifocal pseudophakic eyes. Arq. Bras. Oftalmol..

[10] Fu Y., Kou J., Chen D., Wang D., Zhao Y., Hu M., Lin X., Dai Q., Li J., Zhao Y.E. (2019). Influence of angle kappa and angle alpha on visual quality after implantation of multifocal intraocular lenses. J. Cataract Refract. Surg..

[11] Piracha A.R. (2016). Using Angle Alpha in Premium Iol Screening. Cataract Refract. Surg. Today.

[12] Berdahl J.P., Waring G.O. (2012). Match right lens to patient needs: 10 objective measurements can improve multifocal IOL implantation outcomes [monograph on the internet]. Ophthalmol. Times.

[13] Holladay J.T. (2019). Apparent chord mu and actual chord mu and their clinical value. J. Cataract Refract. Surg..

[14] Karhanová M., Marešová K., Pluháček F., Mlčák P., Vláčil O., Šín M. (2013). Význam úhlu kappa pro centraci multifokálních nitroočních čoček. Ces. Slov. Oftalmol..

[15] Qi Y., Lin J., Leng L., Zhao G., Wang Q., Li C., Hu L. (2018). Role of angle κ in visual quality in patients with a trifocal diffractive intraocular lens. J. Cataract Refract. Surg..

[16] Fernández J., Rodríguez-Vallejo M., Martínez J., Tauste A., Piñero D.P. (2018). Biometric Factors Associated with the Visual Performance of a High Addition Multifocal Intraocular Lens. Curr. Eye Res..

[17] Wang R., Long T., Gu X., Ma T. (2020). Changes in angle kappa and angle alpha before and after cataract surgery. J. Cataract Refract. Surg..

[18] Dapena I., Moutsouris K., Droutsas K., Ham L., Van Dijk K., Melles G.R.J. (2011). Standardized “no-touch” technique for descemet membrane endothelial keratoplasty. Arch. Ophthalmol..

[19] Rodríguez-Vallejo M., Piñero D.P., Fernández J. (2019). Avoiding misinterpretations of Kappa angle for clinical research studies with Pentacam. J. Optom..

[20] Karhanová M., Pluháček F., Mlčák P., Vláčil O., Šín M., Marešová K. (2015). The importance of angle kappa evaluation for implantation of diffractive multifocal intra-ocular lenses using pseudophakic eye model. Acta Ophthalmol..

[21] Prakash G., Prakash D.R., Agarwal A., Kumar D.A., Jacob S. (2011). Predictive factor and kappa angle analysis for visual satisfactions in patients with multifocal IOL implantation. Eye.

[22] Vasiliauskaitė I., Oellerich S., Ham L., Dapena I., Baydoun L., van Dijk K., Melles G.R.J. (2020). Descemet Membrane Endothelial Keratoplasty: Ten-Year Graft Survival and Clinical Outcomes. Am. J. Ophthalmol..

[23] Parekh M., Baruzzo M., Favaro E., Borroni D., Ferrari S., Ponzin D., Ruzza A. (2017). Standardizing descemet membrane endothelial keratoplasty graft preparation method in the eye bank-experience of 527 descemet membrane endothelial keratoplasty tissues. Cornea.

[24] Chaurasia S., Price F.W., Gunderson L., Price M.O. (2014). Descemet’s membrane endothelial keratoplasty: Clinical results of single versus triple procedures (combined with cataract surgery). Ophthalmology.

[25] Price M.O., Pinkus D., Price F.W. (2020). Implantation of Presbyopia-Correcting Intraocular Lenses Staged After Descemet Membrane Endothelial Keratoplasty in Patients with Fuchs Dystrophy. Cornea.

[26] Hashemi H., KhabazKhoob M., Yazdani K., Mehravaran S., Jafarzadehpur E., Fotouhi A. (2010). Distribution of angle kappa measurements with Orbscan II in a population-based survey. J. Refract. Surg..

[27] Mahr M.A., Simpson M.J., Erie J.C. (2020). Angle alpha orientation and magnitude distribution in a cataract surgery population. J. Cataract Refract. Surg..

[28] Arnalich-Montiel F., Pérez-Sarriegui A., Lauzirika G., Porrua L., Hernández-Verdejo J.L. (2017). Pupillary Abnormalities in Descemet Membrane Endothelial Keratoplasty after Nearly Full Tamponade. Cornea.

[29] Stanzel T.P., Ersoy L., Sansanayudh W., Felsch M., Dietlein T., Bachmann B., Cursiefen C. (2016). Immediate Postoperative Intraocular Pressure Changes After Anterior Chamber Air Fill in Descemet Membrane Endothelial Keratoplasty. Cornea.

[30] Frings A., Druchkiv V., Pose L., Linke S.J., Steinberg J., Katz T. (2019). Analysis of excimer laser treatment outcomes and corresponding angle κ in hyperopic astigmatism. J. Cataract Refract. Surg..

[31] Park C.Y., Oh S.Y., Chuck R.S. (2012). Measurement of angle kappa and centration in refractive surgery. Curr. Opin. Ophthalmol..

[32] Moshirfar M., Hoggan R.N., Muthappan V. (2013). Angle Kappa and its importance in refractive surgery. Oman J. Ophthalmol..

[33] Garzón N., García-Montero M., López-Artero E., Albarrán-Diego C., Pérez-Cambrodí R., Illarramendi I., Poyales F. (2020). Influence of angle κ on visual and refractive outcomes after implantation of a diffractive trifocal intraocular lens. J. Cataract Refract. Surg..

[34] Kanellopoulos A.J., Asimellis G., Georgiadou S. (2015). Digital pupillometry and centroid shift changes after cataract surgery. J. Cataract Refract. Surg..

[35] Miháltz K., Vécsei-Marlovits P.V. (2021). The impact of visual axis position on the optical quality after implantation of multifocal intraocular lenses with different asphericity values. Graefe’s Arch. Clin. Exp. Ophthalmol. = Albr. von Graefes Arch. fur Klin. und Exp. Ophthalmol..

[36] Grzybowski A., Eppig T. (2021). Angle alpha as predictor for improving patient satisfaction with multifocal intraocular lenses?. Graefe’s Arch. Clin. Exp. Ophthalmol. = Albr. von Graefes Arch. fur Klin. und Exp. Ophthalmol..

